# Incorporating heterogeneous lacunary Keggin anions as efficient catalysts for solvent-free cyanosilylation of aldehydes and ketones

**DOI:** 10.1038/s41598-022-15831-1

**Published:** 2022-07-07

**Authors:** Masoume Malmir, Majid M. Heravi, Zahra Yekke-Ghasemi, Masoud Mirzaei

**Affiliations:** 1 Department of Chemistry, Faculty of Physics and Chemistry, Alzahra University, Vanak, PO Box 1993891176, Tehran, Iran; 2 Department of Chemistry, Faculty of Science, Ferdowsi University of Mashhad, Mashhad, 9177948974 Iran

**Keywords:** Chemistry, Catalysis, Catalyst synthesis, Heterogeneous catalysis

## Abstract

Polyoxometalates (POMs) as efficient catalysts can be used a wide range of chemical transformations due to their tunable Brønsted/Lewis-acidity and redox properties. Herein, we reported two hybrid and heterogeneous lacunary Keggin catalysts: (TBA)_7_[PW_11_O_39_] (**TBA-PW**_**11**_**)** and (TBA)_8_[SiW_11_O_39_]·4H_2_O (**TBA-SiW**_**11**_**)** (TBA^+^: tetrabutylammonium) in which [XW_11_O_39_]^n−^ anions were coated by TBA^+^ cations. In this form, TBA^+^ can easily trap reactants on the surface of the catalysts and increase the catalytic reaction. Therefore, the catalytic performance of both POMs was tested in cyanosilylation of numerous compounds bearing-carbonyl group and trimethylsilyl cyanide under solvent-free conditions. **TBA-PW**_**11**_ is more effective than **TBA-SiW**_**11**_, conceivably due to the higher Lewis acidity of the P than the Si center and to the higher accessibility of the metal centers in the framework structure. Noteworthy, the recyclability and heterogeneity of both POMs catalysts were also examined, and the results confirmed that they remain active at least after three recycling procedures.

## Introduction

The cyanosilylation reaction (CYSR) is an imperative C–C bond-forming that provides access to many chemicals, containing an extensive range of biological compounds and poly-functionalized building blocks^1,2^. Cyanohydrin, as a known organic synthon, plays an essential role in chemistry and biology. Cyanohydrins are widely used for synthesizing various α-hydroxy compounds, which are mainly synthesized by the trimethylsilyl cyanide (TMSCN) toward CYSR using heterogeneous and homogeneous catalysts including organocatalyst, Lewis acid, base catalyst^3–12^ and POMs^13–18^. The employ of TMSCN as a CN source is more popular than other methods due to avoiding the unstable and toxic hydrogen cyanide (HCN) in cyanohydrins synthesis. In light of eco-friendly procedures, developing a safe, clean, and powerful pathway, which can efficiently catalyze CYSR, is an imperative area of present‐day research^19^. Although numerous papers have been reported in this field, some of which had limitations, such as, tedious separation and recycling problems, the presentation and promotion of a suitable method for synthesizing cyanohydrins are needed. Thus, investigating a mild and effective heterogeneous catalytic system for the CYSR of carbonyl compound and TMSCN under solvent-free conditions is still highly desirable.

Polyoxometalates (POMs) are known as anionic inorganic compounds with diverse structures and fascinating applications that arise from their various physicochemical properties and can be used in various fields such as magnetism^20^, medicine^21^, catalysis^22–24^ analytical chemistry^25,26^. POMs as a catalyst have several benefits such as redox and acid–base properties that can be fine-tuned by changing the chemical structures and compositions, POMs are oxidative and thermally stable compounds compared with organometallic complexes, and the catalytically active sites of POMs can be precisely controlled with an appropriate combination of transition metals and lacunary POMs as inorganic ligands. Generally, POMs can contain Brønsted and Lewis acid sites and are also referred as bifunctional catalysts due to the incorporation of redox and Lewis centers in one unit^27,28^. In addition, the anionic charge of POMs is delocalized over the oxygen atoms, therefore, surface basic oxygen atoms can act as Brønsted base and/or act as a Lewis base (nucleophile)^29,30^.

Up to now, Keggin with the total formula [XM_12_O_40_]^n–^ (X = hetero atom, M = addenda atom) is the most well-studied type of POMs due to its unique structure and stability under different conditions. Lacunary Keggin can be prepared by removing one or more addenda atoms from the complete structure. The removal of one, two, or three addenda metals will respectively lead to the formation of mono-, di-, and tri-lacunary species. This operation is mainly controlled by the variation of the pH of the solution to tailor the desired structure. Lacunary Keggin possesses a higher negative charge than its complete form (anionic charge of the POM)/(number of non-hydrogen atoms of the POM)^22^. Up to now, Keggin-type POMs have been widely used as an oxidation catalyst^31^, and those containing P as the heteroatom showed higher catalytic activity. This behavior can be explained with different electronegativity of the heteroatoms, (P (2.19) > Si (1.90) > Al (1.61)). Fully, the lower electronegativity of the heteroatom leads the more polarized bond between this atom and the oxygen bridging atom as well as the addenda metal sites, resulting to an increase in the basicity of the POM^28,32,33^.

POMs are normally soluble in both water and polar organic solvents and counter-cations play an essential role in the solubility of POMs. For example, POMs with small cations such as Na^+^ or H^+^ are highly soluble in water and other polar organic solvents. On the other hand, POMs with large cations such as Cs^+^, tetrabutylammonium (TBA^+^), or dimethyldioctadecylammonium (DODA^+^) are insoluble in water and exhibit the low absorptive capacity for polar molecules. Therefore, the later groups can be categorized as heterogeneous catalysts^34,35^.

**Figure 1:**
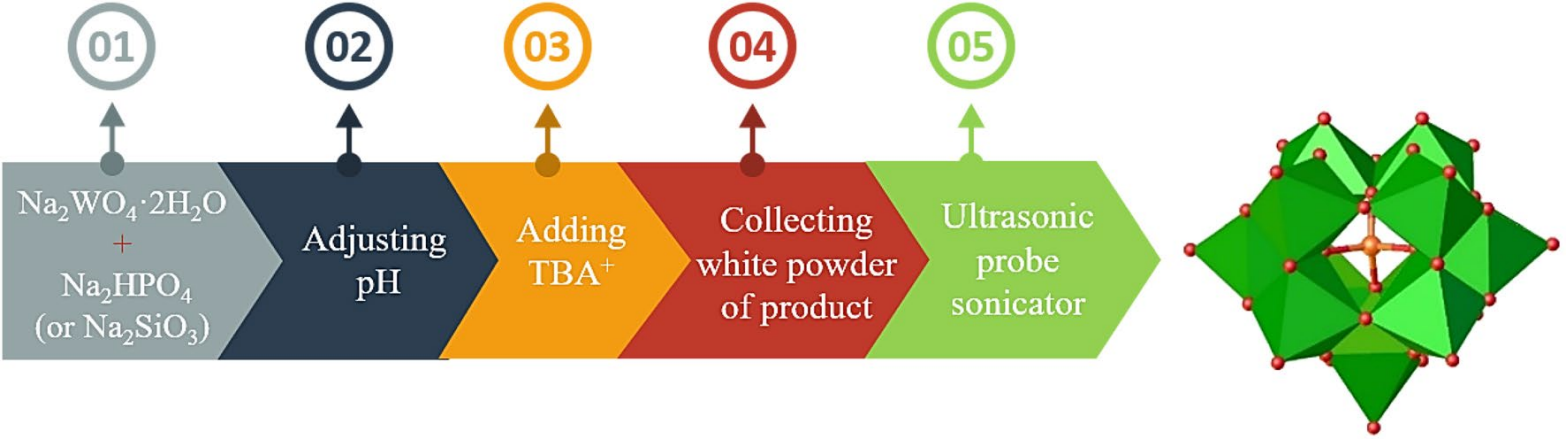
The illustration of the preparation route of nano-sized mono-lacunary Keggin anion. Color code = W: green polyhedral, P or Si: orange, and O: red.

Following our attempt to investigate the synthesis and utility of POM-based catalysts for promoting organic transformations^20–22,24–26,36,37^ and applying heterogeneous nano-catalysts invaluable organic reactions^38–44^ herein, we wish to present two nano-sized organic–inorganic hybrid systems (TBA)_7_[PW_11_O_39_] (**TBA-PW**_**11**_**)** and (TBA)_8_[SiW_11_O_39_]·4H_2_O (**TBA-SiW**_**11**_**)** as heterogeneous catalysts to promote the synthesis of phenyl-trimethylsilanyloxy-acetonitrile derivatives by using aldehydes and ketones using TMSCN under solvent-free conditions (Fig. 1).

## Results and discussion

### Synthesis and characterization of catalysts

**Figure 2:**
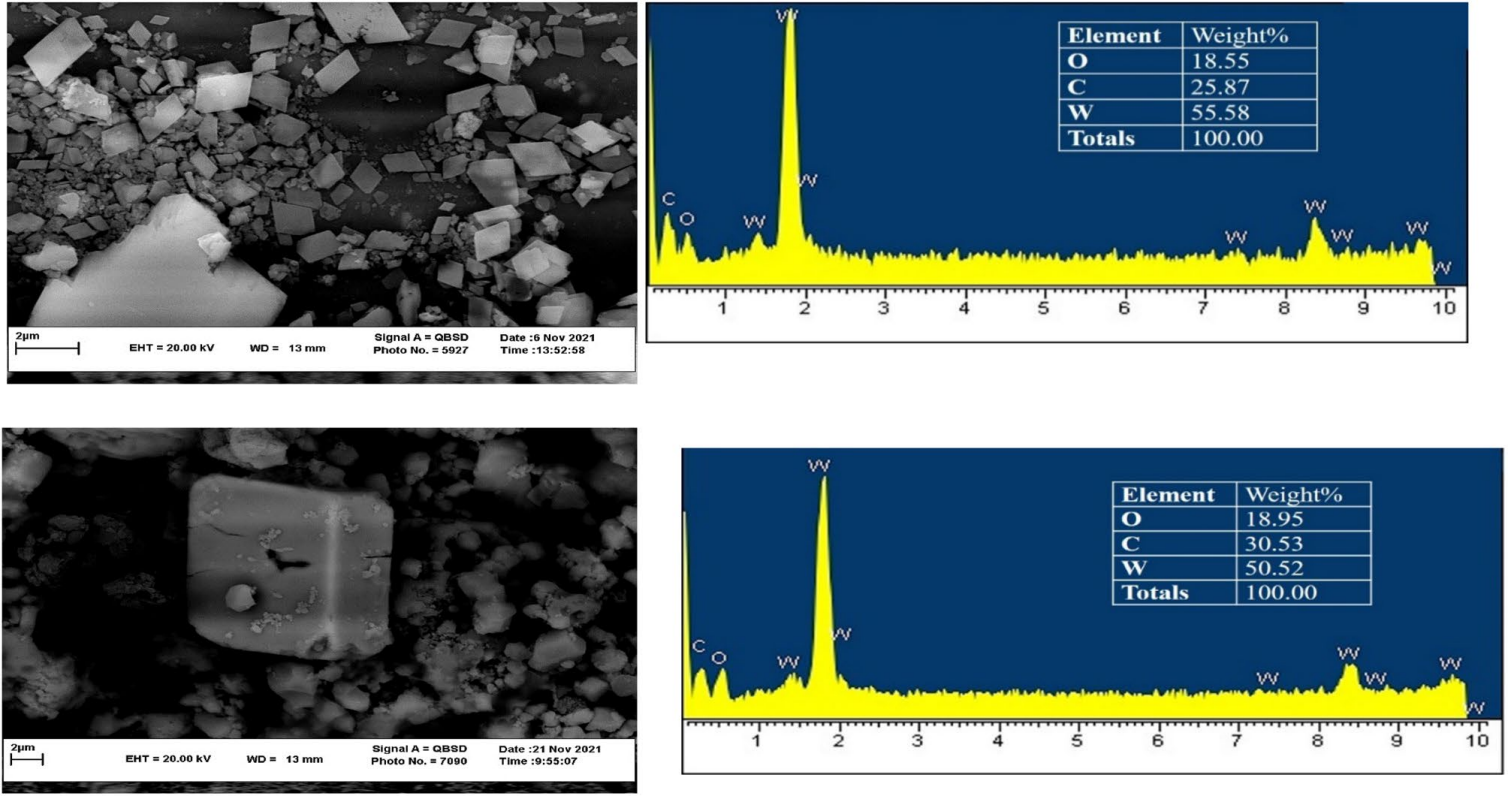
SEM images and EDS spectra of (up) **TBA-PW**_**11**_ and (down) **TBA-SiW**_**11**_ nano-catalysts.

In this study, two heterogeneous nanocatalysts, **TBA-PW**_**11**_ and **TBA-SiW**_**11**_, were obtained by top-down approach with ultrasonic method upon 15 min of sonication. According to the SEM images, morphologies of **TBA-PW**_**11**_ and **TBA-SiW**_**11**_ can consider as rhombic and cubic (Fig. 2). Furthermore, the presence of O, C, and W, in the nanocatalysts is confirmed by the EDS spectrum (Fig. 2).

**Figure 3:**
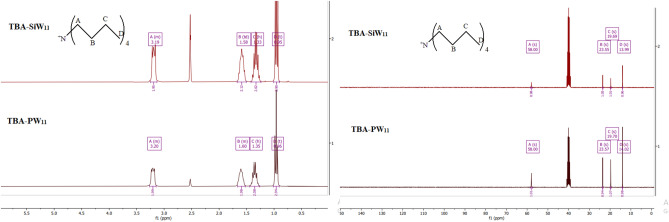
^1^HNMR (left) and ^13^CNMR (right) spectra of **TBA-PW**_**11**_ and **TBA-SiW**_**11**_**.**

Also, there are several examples that the outer surface of anionic POMs can be surrounded by organic cations (like TBA^+^)^45,46^ and in the case of our catalysts, ^1^H NMR and ^13^C NMR spectra provide clear and direct evidence for the presence of TBA^+^. For example, in the ^1^H NMR, three peaks located separately around 1.35, 1.60, and 3.20 ppm can be assigned to the CH_2_ of TBA^+^ and the CH_3_ group located at 0.96 ppm (Fig. 3). Also, The ^31^P NMR spectrum of **TBA-PW**_**11**_ (Fig. S1) was in the normal range of diamagnetic phosphotungstate and showed one peak at − 13.21 ppm, corresponding to the P atoms in the lacunary anion^47^.

The IR spectra of POMs include characteristic metal–oxygen stretching vibrations that occur in the specific region (generally sharp bands between 400−1000 cm^−1^). The characteristic strong bands for X–O, W–O_t_, W–O_b_ and W–O_c_ stretching vibrations of nanocatalysts are shown in Table 1 and Fig. S2, which can approve the exact structures of the final catalysts. Moreover, absorptions in the range 2873–2961 cm^–1^ correspond to the C–H stretching vibrations of TBA^+^. Specific bands at around 1700 and 3300 cm^-1^ assigned to water molecules^48^.

**Table 1:** Representation of important absorption bands (cm^−1^) for **TBA-PW**_**11**_ and **TBA-SiW**_**11**_ heterogeneous and nano catalysts.

Compound	ν_as_ (X–O_a_)	ν_s_ (X–O_a_)	ν_as_ (W–O_t_)	ν_as_ (W–O_b_) and ν_as_ (W–O_c_)	ν (C–H)
TBA-PW11	1053	517	957	888, 805	2873–2961
Nano-sized TBA-PW11	1059	516	958	888, 812	2873–2961
TBA-SiW_11_	1061	532	966	920, 804	2873–2960
Nano-sized TBA-SiW11	1059	531	966	920–801	2872–2961

### Catalytic activity

To extend the catalytic capacity of **TBA-PW**_**11**_ and **TBA-SiW**_**11**_ catalysts, herein, we study the achievement of both catalysts for the CYSR obtained from two reaction pathways. For this purpose, the reaction between 1 mmol of benzaldehyde (BA) and TMSCN (2 mmol) was selected as a model reaction and performed using **TBA-SiW**_**11**_ and **TBA-PW**_**11**_ (2 mol%), separately, without any solvents. Gratifyingly, the desired products were isolated and obtained in 65% and 96%, respectively, after 45 min. Encouraged by these results, different reaction conditions such as temperature, catalyst amount, and solvent were optimized (Table S1). In this context, the selected reaction was accomplished at different temperatures (r.t., 65 °C, and 90 °C) under S.F., which the CYSR led to the highest yield at 65 °C. Moreover, only 55% or 40% of product yield upon performing the reaction at r.t. (Table S1, entries 1 and 2), using **TBA-PW**_**11**_ or **TBA-SiW**_**11**_ as catalysts, respectively. Increasing the reaction temperature from 65 °C to 90 °C did not improve the reaction yields (Table S1, entry 14). After that, the effect of various solvents, such as CHCl_3_, MeOH, toluene, and THF, was examined on the CYSR. As evident, S.F. conditions demonstrate the higher activity, leading to a corresponding product yield of 96% or 65% for **TBA-PW**_**11**_ and **TBA-SiW**_**11**_, respectively (Table S1, entries 3 and 4), while, using other solvents was not suitable for CYSR due to the production of low-yield products in the range of trace to 78% (Table S1, entries 10–13). For finding the optimum catalyst amount, the CYSR was performed over a different amount of both catalysts from 1 to 3 mol%, and significant development of the product yield was observed from 45 to 96% for **TBA-PW**_**11**_ or from 35 to 80% for **TBA-SiW**_**11**_ (Table S1, entries 3, 4 and 6–9), noteworthy, the use of 3 mol% of both catalysts had no effect on improving the CYSR. Therefore, the best catalyst amount was achieved as 2 mol%. Finally, a blank test using BA and TMSCN without any solvent and catalyst at 65 °C was accomplished and resulted in only a trace yield of the final product after 3 h (Table S1, entry 5). Therefore, the best conditions for promoting CYSR of the BA and TMSCN concern 2 mol% of the **TBA-PW**_**11**_ as the best catalyst at 65 °C without any solvent.

Following, the performance of **TBA-PW**_**11**_ as the best catalyst was tested towards different substituted ketones and aldehydes (Table 2). As tabulated, various aldehyde-containing compounds with different electron densities could tolerate these reactions to provide the desired products in high yields. Generally, the aldehydes bearing electron‐withdrawing groups, for instance, nitro, bromo, and chloro groups, show the excellent activity with the highest yields, and the potential of the *-para* position was significant in advancing the reaction to the *ortho* and *meta* positions (Table 2, entries 1–4 and 7–9). In contrast, the aldehyde bearing electron-donating groups (methyl and methoxy groups) exhibit lower yields in longer reaction time (Table 2, entries 5 and 6). Moreover, ketone compounds with electron‐withdrawing groups show moderate to good reactivity and produce the corresponding phenyl-trimethylsilanyloxy-acetonitrile derivatives (Table 2, entries 10–13), being also above the corresponding yields for the ketones with electron‐donor substituents. However, the excellent reactivity of aldehydes compared to ketones is quite apparent. These behaviors are in agreement with the predictable effect of the substituent on the electrophilic character of the carbonyl groups to undergo attack by the -cyano group of TMSCN.

**Table 2:** CYSR of different substituted carbonyl compounds with TMSCN with catalyst **TBA-PW**_**11**_**.**


Entry	R	X	Products	Yield^a^ (%)	Time (min)
1	H	H	**2a**	96	45
2	*p*-NO_2_	H	**2b**	98	45
3	*m*-NO_2_	H	**2c**	88	70
4	*o*-NO_2_	H	**2d**	90	55
5	*p*-Me	H	**2e**	85	90
6	*p*-OMe	H	**2f**	88	80
7	*p*-OH	H	**2g**	78	75
8	*p*-Cl	H	**2h**	84	80
9	*p*-Br	H	**2i**	88	65
10	H	Me	**2j**	90	90
11	*p*-NO_2_	Me	**2k**	91	75
12	*p*-Me	Me	**2l**	80	110
13	*p*-OMe	Me	**2m**	82	105

Reaction conditions: aldehyde (1 mmol), TMSCN (2 mmol), 2 mol% of TBA-PW_11_ at 65 °C at S.F.

^a^Isolated yields.

Aiming to evaluate the benefits of this study, the catalytic activity of the **TBA-PW**_**11**_ towards the CYSR of BA with TMSCN was compared with other literature, as shown in Table 3. As tabulated, **TBA-PW**_**11**_ shows high efficiency, in a shorter time, under solvent‐free conditions compared to **TBA-SiW**_**11**_ and other reported catalysts (Yield of 96%, in S.F. at 65 °C after 45 min, Table 3, entry 6).

**Table 3:** A comparison of catalytic activity of different reported coordination polymers in the CYSR of BA with TMSCN.

Entry	Catalysts (amount)	Reaction conditions (solvent/temperature (°C))	Time (h:min)	Yield (%)	Ref.
1^a^	[Zn(μ‐1κ*O*^1^:1κ*O*^2−^L)(H_2_O)_2_]_n_∙n(H_2_O) (2 mol%)	S.F./50/MW	01:30	97	^50^
2^b^	[Gd_2_(bpt)_2_(H_2_O)_2_]·(DMF)_2_(H_2_O)_6_ (2.5 mol%)	S.F./50/N_2_	02:00	99.3	^51^
3	MIL-101 (Cr) (0.3 mol%)	S.F./r.t	04:00	96	^52^
4^c^	{[Cu_2_(bpy)(H_2_O)_5.5_]_2_[H_2_W_11_O_38_]·3H_2_O·0.5CH_3_CN} (2 mol%)	CH_3_CN/r.t./N_2_	24:00	98.1	^53^
5^d^	[Cu_2_(H_2_O)_2_(*μ*-H_2_L^3^)(*μ*-L^3^)]n (5 mol%)	MeOH/r.t	04:00	94.9	^6^
6^e^	[Zn_4_(*μ*-OH)_2_(1k*O*:2k*O*-HL^3^)4(k*O*-HL^3^)_2_(H_2_O)_4_] (4 mol%)	MeOH/r.t	03:00	79	^54^
7	P(MeNMCH_2_CH_2_)_3_ N (10 mol%)	THF/0 °C	01:00	67	^55^
8^f^	[Ag_4_(apym)_4_SiW_12_O_40_)]_n_ (0.1 mol%)	S.F./r.t./N_2_	04:00	96.2	^15^
9^g^	H[Ni(en)_3_]_5_[VNb_12_O_40_(VO)_2_]ˑ15H_2_O (1 mol%)	S.F./r.t./N_2_	01:20	89.29	^18^
10	TBA-SiW_11_ (2 mol%)	S.F./65 °C	0:45	65	This work
11	TBA-PW_11_ (2 mol%)	S.F./65 °C	0:45	96	This work

Reaction conditions: A mixture of BA, TMSCN, and catalyst in solvents at different temperatures.

^a^L: 5‐{(pyren‐4‐ylmethyl)amino}isophthalate.

^b^bpt: biphenyl-3,4′,5-tricarboxylate.

^c^ bpy: bipyridine.

^d^H_3_L^3^: 2-(2-(4,4-dimethyl-2,6-dioxocyclohexylidene)hydrazinyl)terephthalic acid.

^e^HL^3^: 2-(2-(2,4-dioxopentan-3-ylidene)hydrazineyl)benzoate.

^f^apym: 2-aminopyrimidine.

^g^en: ethylenediamine.

**Figure 4:**
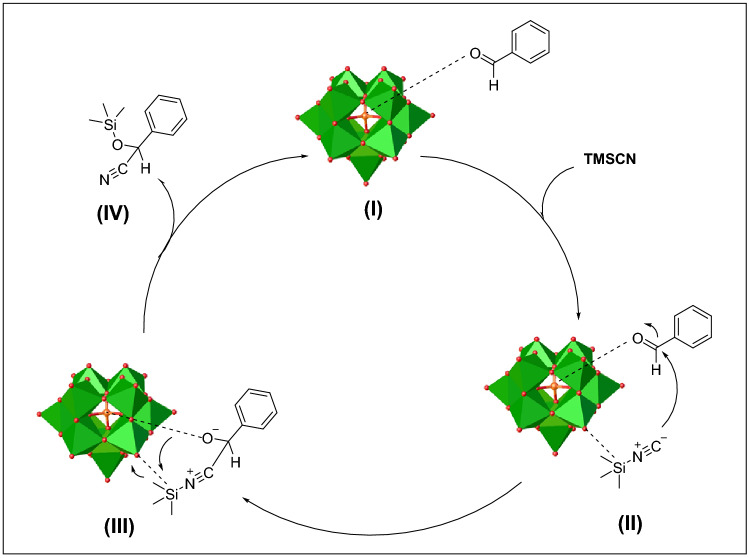
Proposed mechanism for the CYSR of carbonyl compounds catalyzed by lacunary Keggin **TBA-PW**_**11**_ or **TBA-SiW**_**11**_.

Commensurate with the experimental results and previously reported literatures, a possible CYSR mechanism is proposed and illustrated in Fig. 4^8,49^. First, the carbonyl group in BA was activated by the coordinatively central P or Si atoms in catalysts (**I**) to nucleophilic attack of CN group in TMSCN (**II**). Finally, with the migration of the silyl group to the oxygen of intermediate (**III**), a carbon–carbon bond and then cyanohydrin (**IV**) is formed (Fig. 4). Notably, the products were replaced by BA, and the catalysts were continued to activate the BA in the next catalytic cycle.

## Experimental

### Chemicals and materials

The chemical compounds were purchased from Merck (Darmstadt, Germany, www.merckmillipore.com) and Sigma-Aldrich (St. Louis, MO, USA, www.sigmaaldrich.com) and used with no crystallization or purification. To conduct CYSR, aromatic aldehydes and ketones, TMSCN, toluene, methanol, chloroform, and THF were used.

### Instrumentation

Melting points were determined with an electrothermal 9200 digital melting point apparatus (www.labnet.fi). Infrared (FT-IR) spectra were measured using KBr pellets containing the compounds (400–4000 cm^−1^) with a Bruker Tensor 27 FT-IR spectrometer. Also, the infrared spectra of catalysts were recorded in the range of 4000–400 cm^–1^ on a Thermo Nicolet/AVATAR 370 Fourier transform spectrophotometer (www.thermofisher.com) using KBr discs. A Thermo Finnigan Flash 1112EA elemental analyzer (www.thermofisher.com) was used for elemental analysis (C, H and N) compounds. The Spectro Arcos ICP-OES spectrometer model 76,004,555 in the range of 130–770 nm was measured metal content. ^1^H NMR, ^13^C NMR, and ^31^P NMR spectra were recorded in DMSO-*d*_*6*_ as the solvent on a Bruker FUM-300 spectrometer.

### Preparation of catalysts

First, heterogeneous catalysts (TBA)_7_[PW_11_O_39_] (**TBA-PW**_**11**_) and (TBA)_8_[SiW_11_O_39_]·4H_2_O (**TBA-SiW**_**11**_) were synthesized and identified by FT-IR, elemental analysis, and NMR spectroscopy. Then, the top-down approach using ultrasonic technique were successfully synthesized the related nanocatalysts and characterized by FTIR and SEM–EDS.

### Synthesis of (TBA)_7_[PW_11_O_39_] (TBA-PW_11_)

First, 72.5 g (0.22 mol) Na_2_WO_4_·2H_2_O and 7.16 g Na_2_HPO_4_·12H_2_O (0.02 mol) were dissolved in 100 mL water, which was heated to 70–80 °C. Dropwise HNO_3_ was added to adjust the pH of the solution to 3.0. The solution was concentrated to half of the initial volume by heating at 80 °C. Then, a hot water solution of TBABr (7 mmol, 7 mL) was added to the above mixture and stirred for a further 30 min. The resulted white precipitates were filtered off, washed twice with water and dried in vacuum.

### Synthesis of (TBA)_8_[SiW_11_O_39_]·4H_2_O (TBA-SiW_11_)

First, Na_2_WO_4_·2H_2_O (182 g, 0.55 mol) is dissolved in 300 mL of boiling distilled water, and a solution of HCI (4 M, 165 mL) is added dropwise (in 30 min) to this. Next, 100 mL solution of sodium metasilicate (1.1 g, 50 mmol) was added quickly to the previous solution, and 50 mL of HCI (4 M) is also added. The pH is about 5 to 6. The solution is kept boiling for 1 h. After cooling to room temperature, the solution is filtered if it is not completely clear. Then, a hot water solution of TBABr (8 mmol, 7 mL) was added to the above solution, which is stirred magnetically for a further 30 min. The white precipitation of the product was collected by filtration and washed twice with water.

### Synthesis of nano-sized TBA-PW_11_ and TBA-SiW_11_

The mixture solution of Ethanol (10 mL), water (15 mL) and **TBA-PW**_**11**_ (or **TBA-SiW**_**11**_) (0.03 g) was subjected to ultrasonication (200 W). After 15 min, nano-sized catalysts were collected by centrifugation and then washed with water (3 × 5 mL) under a vacuum.

### Typical method for the CYSR of carbonyl compounds

In a tube, a mixture of a carbonyl compound (1 mmol), TMSCN (2 mmol), and 2 mol% of **TBA-PW**_**11**_ or **TBA-SiW**_**11**_ was prepared, and it was put in an oil bath. After that, the mixture was heated at 65 °C without any solvent, for the desired time. Upon completion of CYSR, both mentioned catalysts were separated by filtration, and the mixture's solvent was evaporated. Finally, the pure product was dissolved and achieved in CH_2_Cl_2_.

### Catalyst recyclability

Moreover, for examining the heterogeneous nature of the **TBA-PW**_**11**_ and **TBA-SiW**_**11**_, both catalysts separated from the reaction after 20 min and kept the catalyst‐free reaction under a similar environment for 25 min more. After removing the **TBA-PW**_**11**_ and **TBA-SiW**_**11**_ catalysts from the reaction mixture, no noticeable rise in product yield was detected, which verifies the heterogeneous nature of both catalysts. Further, to explore the recyclability of both catalysts, the catalytic activities of the fresh and reused **TBA-PW**_**11**_ and **TBA-SiW**_**11**_ were studied and compared. For this purpose, after completing each reaction cycle, catalysts were separated by simple filtration, washed with EtOH, and dried. As exhibited in Fig. S3, **TBA-PW**_**11**_ and **TBA-SiW**_**11**_ catalysts could be effectively recycled three times. However, the **TBA-SiW**_**11**_ catalyst experienced a significant loss in catalytic activity compared to the **TBA-PW**_**11**_ catalyst.

**Figure 5:**
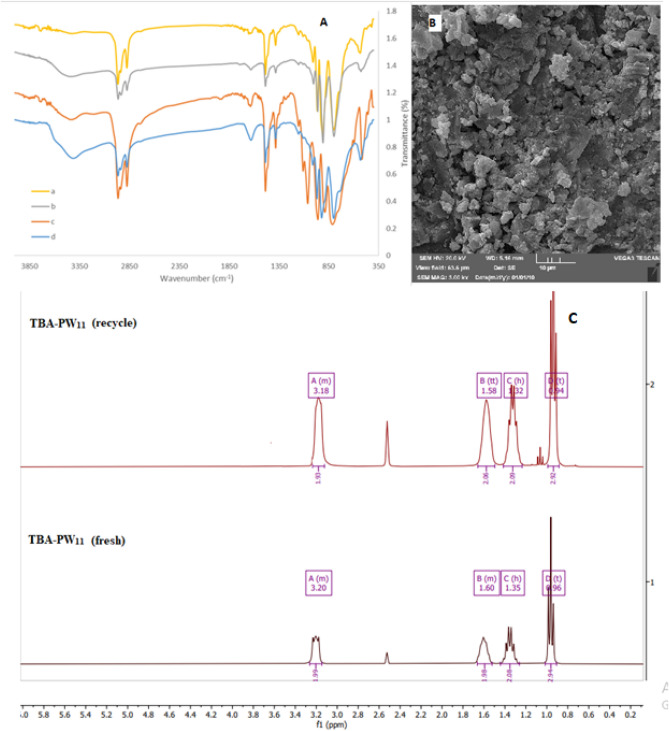
The FTIR analyses of the fresh and recycled **TBA-PW**_**11**_ (a, b) and fresh and recycled **TBA-SiW**_**11**_ (c, d) (**A**), respectively, SEM image of the recycled **TBA-PW**_**11**_ (**B**) and the ^1^HNMR spectra of the fresh and recycled **TBA-PW**_**11**_ (**C**).

Finally, to check the structural integrity, FTIR analysis of the fresh and recycled **TBA-PW**_**11**_ and **TBA-SiW**_**11**_ were recorded. As shown in Fig. 5A, no momentous changes in their patterns were detected. In addition, to elucidate whether the recycling process can result in any change in the catalyst's morphology and catalyst structure, the ^1^HNMR spectra and the SEM images of the recycled **TBA-PW**_**11**_ catalyst were recorded (Fig. 5B,C). These results support that the structure of the **TBA-PW**_**11**_ underwent several reactions was preserved, but some agglomeration is evident.

### Characterization data

#### Spectral data for catalysts

**TBA-PW**_**11**_: Yield: 73% based on W. Anal. Calcd. for C_112_H_252_N_7_O_39_PW_11_: C, 30.75; W, 46.23; N, 2.24; P, 0.71; H, 5.81%. Found: C, 29.82; W, 45.78; N, 2.08; P, 0.67; H, 6.11%. FT-IR (KBr pellet, cm^−1^): 2961, 2937, 2873, 1484, 1461, 1381, 1155, 1106, 1053, 957, 888, 805, 754, 596, 517, 407. ^1^H NMR (*d*_*6*_-DMSO, 300 MHz, RT) [δ, ppm] 0.96 (t, TBA-CH_3_), 1.35 (h, TBA-CH_2_), 1.60 (dq, TBA-CH_2_), 3.20 (m, TBA-CH_2_). ^13^C NMR (*d*_*6*_-DMSO, 300 MHz, RT) [δ, ppm] 14.02, 19.70, 23.57, 58.00 (all singlets). ^31^P NMR (*d*_*6*_-DMSO, 300 MHz, RT) [δ, ppm] (− 13.21) (singlet).

**TBA-SiW**_**11**_: Yield: 68% based on W. Anal. Calcd. for C_128_H_296_N_8_O_43_SiW_11_: C, 32.81; W, 43.15; N, 2.39; Si, 0.60; H, 6.37%. Found: C, 33.15; W, 42.65; N, 2.45; Si, 0.58; H, 6.55%. FT-IR (KBr pellet, cm^−1^): 3410, 2960, 2937, 2873, 2732, 1633, 1484, 1472, 1462, 1380, 1152, 1108, 1061, 1007, 966, 920, 894, 804, 739, 532. ^1^H NMR (*d*_*6*_-DMSO, 300 MHz, RT) [δ, ppm] 0.95 (t, TBA-CH_3_), 1.33 (h, TBA-CH_2_), 1.58 (pd, TBA-CH_2_), 3.19 (m, TBA-CH_2_). ^13^C NMR (*d*_*6*_-DMSO, 300 MHz, RT) [δ, ppm] 13.99, 19.69, 23.55, 58.00 (all singlets).

**Nano-TBA-PW**_**11**_: FT-IR (KBr pellet, cm^−1^): 2961, 2937, 2873, 1484, 1381, 1155, 1108, 1059, 958, 888, 812, 754, 595, 516.

**Nano-TBA-SiW**_**11**_: FT-IR (KBr pellet, cm^−1^): 3414, 2961, 2932, 2873, 1627, 1484, 1381, 1155, 1106, 1059, 966, 920, 801, 738, 531.

## Concluding remarks

In summary, two nano-sized organic–inorganic hybrid systems based on lacunary Keggin **TBA-PW**_**11**_ and **TBA-SiW**_**11**_ as heterogeneous catalysts were synthesized and characterized using a suite of analytical techniques. Due to the coexistence of the high negative charge of the above catalysts, they showed an excellent catalytic effect for cyanosilylation of various aldehydes and ketones, giving the corresponding cyanohydrin trimethylsilyl ethers with high yields in a short time. Notably, both catalysts were heterogeneous, but **TBA-PW**_**11**_ showed higher catalytic activity and recyclability towards the cyanosilylation of aldehydes under S.F. conditions (96%) in comparison with **TBA-SiW**_**11**_. Also, further studies are underway in our laboratory to extend the application of these family catalysts to other coupling reactions.

## Supplementary Information

Supplementary Information.

## Data Availability

All data generated or analysed during this study are included in this published article (and its Supplementary Information files).
