# Supplementary material for: Incorporating heterogeneous lacunary Keggin anions as efficient catalysts for solvent-free cyanosilylation of aldehydes and ketones
Source: Sci Rep. 2022 Jul 7;12:11573. doi: 10.1038/s41598-022-15831-1 (PMC9262904; doi:10.1038/s41598-022-15831-1)
Supplement: Supplementary file 1 — Supplementary Information. [file 41598_2022_15831_MOESM1_ESM.pdf]

Supporting information

Incorporating heterogeneous lacunary Keggin anions as efficient catalysts for solvent-free cyanosilylation of aldehydes and ketones

Masoume Malmir [a], Majid M. Heravi [a,*], Zahra Yekke-Ghasemi [a], Masoud Mirzaei [b,*]

[a] *Department of Chemistry, Faculty of Physics and Chemistry, Alzahra University, PO Box 1993891176, Vanak, Tehran, Iran.*

[b] *Department of Chemistry, Faculty of Science, Ferdowsi University of Mashhad, Mashhad 9177948974, Iran.*

*Corresponding author, Email: mmheravi@alzahra.ac.ir, ORCID number: 0000-0002-9259-0591

*Corresponding author, E-mail: mirzaeesh@um.ac.ir, ORCID number: 0000-0002-7256-4601

-

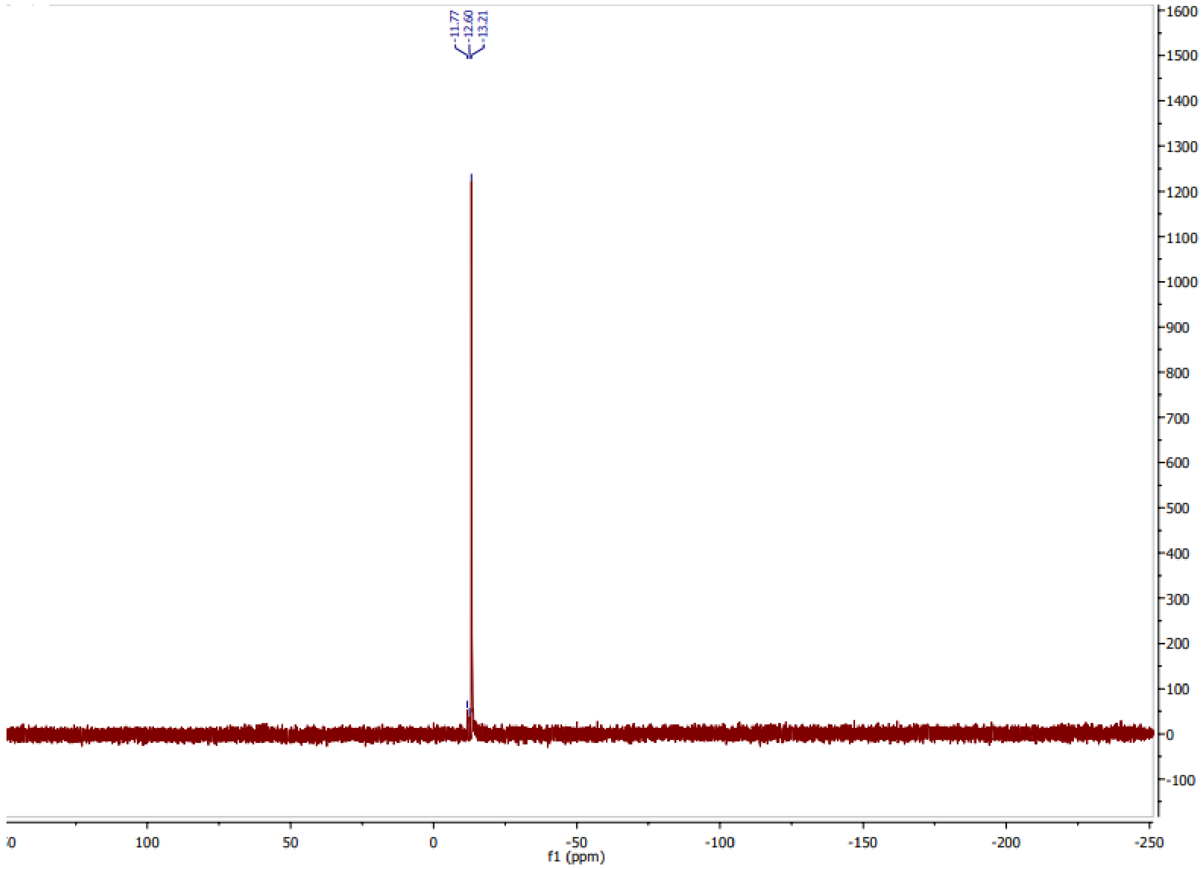

Fig. S1. ^{31}P NMR spectra of **TBA-PW$_{11}$** heterogeneous and nano-catalysts

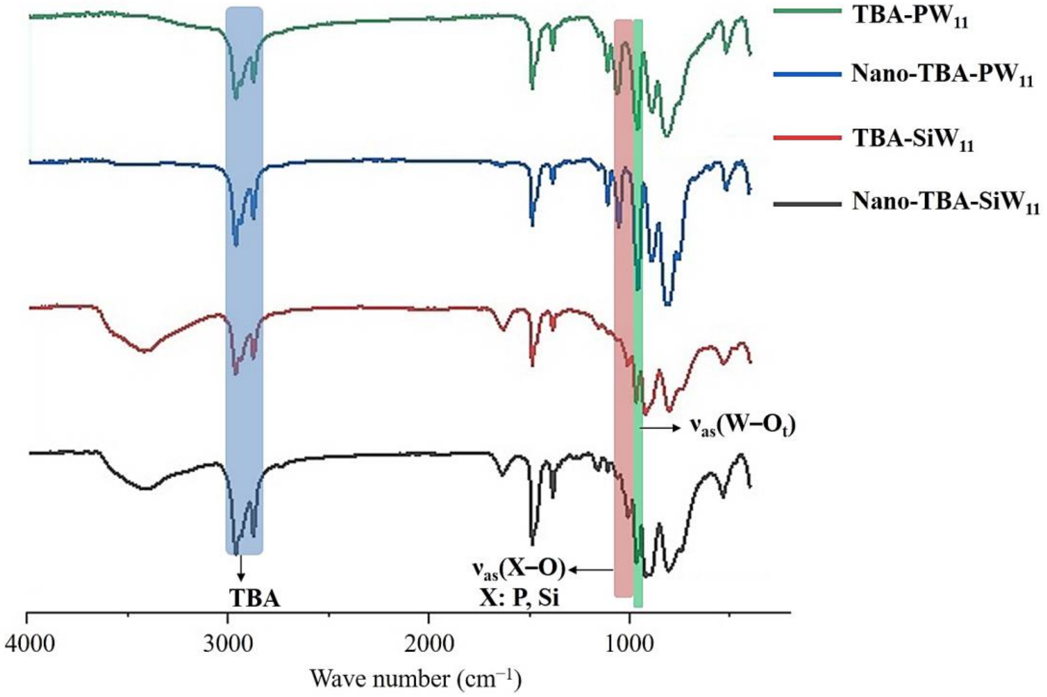

Fig. S2. FTIR spectra of **TBA-PW$_{11}$** and **TBA-SiW$_{11}$** heterogeneous and nano-catalysts.

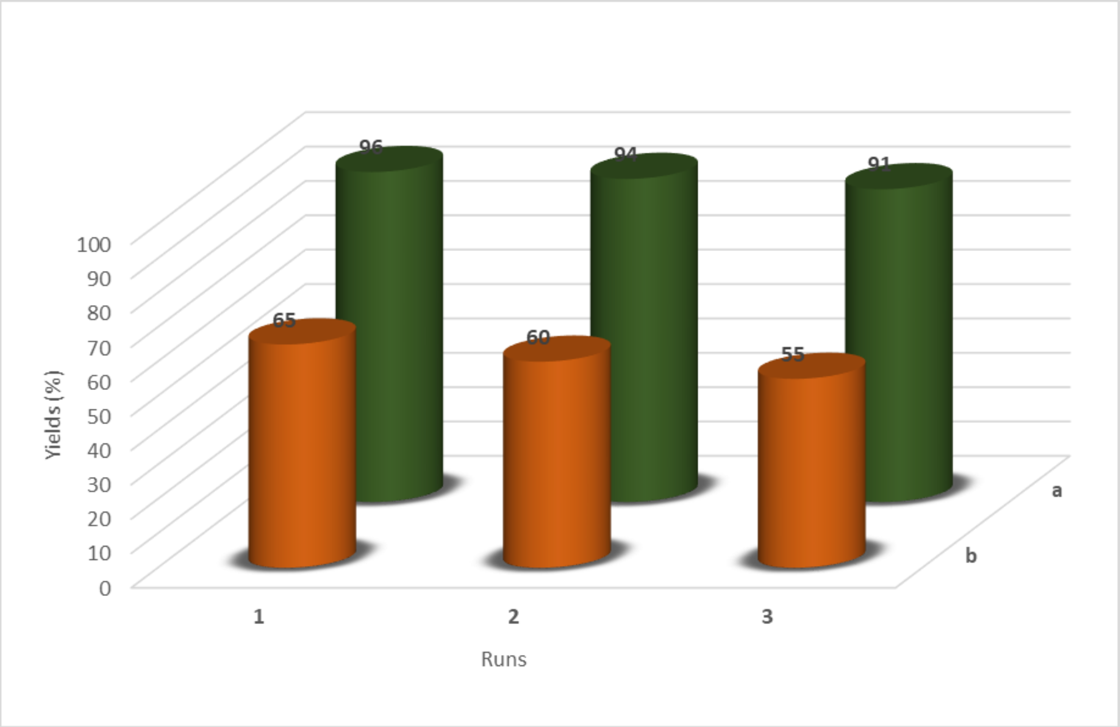

Fig. S3. The recyclability of the **TBA-PW$_{11}$** (green columns) and **TBA-SiW$_{11}$** (orange columns) catalysts in CYSR.

Table S1. CYSR of BA with TMSCN in the presence of **TBA-PW11** or **TBA-SiW11** as catalysts.[a]

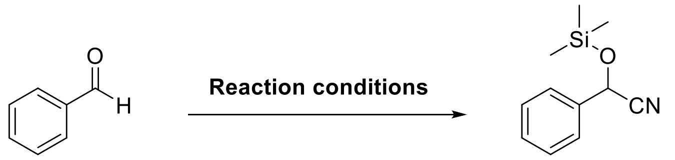

Entry	Catalyst (mol%)	Reaction conditions Temperature C°/ Solvent	Yields (%)	Time (min)
1	TBA-PW_{11} (2)	r.t./ S.F.	55	45
2	TBA-SiW_{11} (2)	r.t./ S.F.	40	45
3	TBA-PW_{11} (2)	65/ S.F.	96	45
4	TBA-SiW_{11} (2)	65/ S.F.	65	45
5	None	65/ S.F.	Trace	125
6	TBA-PW_{11} (1)	65/ S.F.	45	45
7	TBA-PW_{11} (3)	65/ S.F.	90	45
8	TBA-SiW_{11} (1)	65/ S.F.	35	45
9	TBA-SiW_{11} (3)	65/ S.F.	80	45
10	TBA-PW_{11} (2)	65/ THF	65	45
11	TBA-PW_{11} (2)	65/ Toluene	55	45
12	TBA-PW_{11} (2)	65/ MeOH	78	45
13	TBA-PW_{11} (2)	65/ $CHCl_3$	Trace	45
14	TBA-PW_{11} (2)	90/ S.F.	90	45

[a] Reaction conditions: BA (1 mmol), TMSCN (2 mmol).
[b] Isolated yields.
